# Edible optical microcavities for optical barcoding, authentication, and sensing

**DOI:** 10.1038/s41598-026-51128-3

**Published:** 2026-05-07

**Authors:** Abdur Rehman Anwar, Slavko Kralj, Matjaž Humar

**Affiliations:** 1 J. Stefan Institute, Jamova 39, SI-1000 Ljubljana, Slovenia; 2 Jožef Stefan International Postgraduate School, Jamova 39, 1000 Ljubljana, Slovenia; 3 Faculty of Mathematics and Physics, University of Ljubljana, Jadranska 19, SI-1000 Ljubljana, Slovenia; 4 CENN Nanocenter, Jamova 39, SI-1000 Ljubljana, Slovenia

**Keywords:** Edible materials, Whispering gallery modes, Optical barcodes, Physical unclonable functions, Sensors, Materials science, Optics and photonics

## Abstract

The safety, authenticity, and traceability of food and pharmaceutical products are critical challenges, especially in the context of widespread counterfeiting. Most existing anti-counterfeiting and sensing technologies are applied externally to packaging, making them vulnerable to tampering. A more robust approach is to incorporate them directly into edible products. Whispering-gallery modes (WGMs), highly sensitive optical resonances supported by spherical microcavities, provide a promising platform due to their environmental responsiveness and spectral uniqueness. Here, we demonstrate edible WGM microcavities based on chlorophyll-coated silica microspheres. Monodisperse microspheres exhibit stable WGM resonances under continuous-wave laser excitation, with quality factors ranging from 2,000 to 10,000. Individual microsphere diameters are determined with a precision of approximately 40 nm. When embedded in low-refractive-index agarose matrices, the microspheres generate size-specific spectral signatures that function as optical barcodes and remain stable for at least six months. Based on intrinsic size variations, we further demonstrate edible physical unclonable functions (PUFs) with stable and unique optical identifiers. In addition, the microcavities enable sugar concentration measurements with uncertainties as low as 0.23 percentage points and pH sensing with an accuracy of approximately 0.3 pH units, when embedded in a pH-responsive hydrogel. These results establish edible WGM microcavities as a multifunctional platform for secure labeling, sensing, and anti-counterfeiting in consumable products.

## Introduction

Food and pharmaceutical counterfeiting and the degradation of their quality are critical challenges that directly impact consumer health and global trade^1,2^. In our previous paper, we demonstrated for the first-time lasers made completely out of edible substances and their potential use as barcodes and sensors^3^. Specifically, we demonstrated whispering gallery modes (WGMs) and Fabry Pérot (FP) edible lasers.

However, for real-life applications, this technology has to be improved. For example, lasing requires a pulsed laser excitation, which is bulky and expensive. The FP cavity laser is labor-intensive to assemble and cannot be operated under (continuous-wave) CW laser excitation. Further, monodisperse microcavities were only made from oil in the form of droplets, which were stable only in water. However, for the barcodes to be usable in any product, including oils, honey, or solid products, they have to be in the form of solid microbeads. For practical applications, they also need to be manufactured cheaply on a mass scale.

Here, we optimize WGMs microcavities for various applications. WGMs are optical resonances that occur when light is confined within a transparent spherical object via total internal reflection. As light continuously circulates along the inner surface of the cavity, it undergoes constructive interference, resulting in well-defined optical modes^4^. WGMs can be excited by coupling light into the spherical cavity through an evanescent field or by doping the cavity with a gain medium, such as fluorescent dyes^5^. Typically, WGM cavities are made up of solid spheres or liquid droplets and require immersion in a medium with a lower refractive index^6,7^. Due to the emission of a characteristic spectrum and their extreme sensitivity to cavity size, shape, and refractive index variations, WGM microspheres have gained a great deal of attention for use as spectral barcodes and sensors^8,9^.

For barcoding using WGMs, two options are available: either to use random sizes and generate random barcodes, or to fabricate well-defined sizes to encode predefined information into the barcode. Random barcodes are generated through inherent variations in microcavity size (polydispersity) that occur during the fabrication process, resulting in each one having a unique and practically irreproducible spectral signature, which has also been observed in clusters of WGM microspheres^10^. This uniqueness is valuable for anti-counterfeiting applications. Moreover, random barcoding allows the production of a large number of unique barcodes^11–13^. Due to their inherent randomness, these barcodes are unclonable and can serve as physical unclonable functions (PUFs). Each product carries a unique tag that is initially read and stored in a database. Later, the tag can be re-read and verified with the database.

On the other hand, predefined barcodes require the generation of microcavities of several well-defined and non-overlapping sizes, and then mixing only the selected sizes to produce a unique barcode. Only a few demonstrations of predefined WGM barcodes have been reported, such as edible WGM microlasers of well-defined sizes by microfluidics^3^. Furthermore, microdroplets and nanobeads were produced at the tip of the microcapillary under controlled pressure, which achieved higher information capacity^14^, but with comparatively slow fabrication. Overall, random barcodes can be readily produced in bulk with minimal complexity. In contrast, predefined barcodes often require microfluidic or high-precision fabrication techniques, which make them comparatively slow and limited in throughput.

Monodisperse beads of various materials can be produced on a large-scale by bulk methods. For example, polystyrene and other polymer beads such as PMMA are usually produced by emulsion or dispersion polymerization, where monomers are polymerized in the presence of stabilizers and surfactants, which allows the precise control over size and uniformity^15,16^. Melamine formaldehyde beads are produced via polycondensation reaction of melamine and formaldehyde by controlling pH and temperature, resulting in the formation of uniform and stable beads^17^.

However, most of these are not edible, making them unsuitable for barcoding edible products. Different microspheres based on natural proteins and polysaccharides such as cellulose, starch, pectin, bovine serum albumin, and curcumin^18–21^ have been made. However, these can not be produced in a monodisperse form in bulk quantities.

There is, however, one material, namely silica, that is both edible and can be used to make monodisperse beads in large quantities via the classical Stöber method^22^. Silica is widely used in the food industry, and it is approved as a food additive in the European Union (E551). It is commonly used as an anti-caking agent, and stabilizer^23^. Silica is also recognized as safe (GRAS) by the US Food and Drug Administration (FDA). Apart from the food industry, silica has been explored in drug delivery and biosensing applications^24^. Importantly, fluorescent silica microspheres have also been reported, in which dyes are either encapsulated or coated on the surface; however these dyes are generally not edible or food grade^25,26^.

WGM microcavities are also well known for sensing applications^27^. Microspheres coupled with fibers have been demonstrated with high quality factors, making them suitable for precise sensing applications^28–30^. However, their direct integration into consumables for continuous monitoring remains challenging, which limits their applicability in a practical environment. Stand alone WGM microcavities in the form of probes have been used to measure refractive index, force, temperature^31–34^. In some cases the microspheres are functionalized to bind specific molecules, or coated with a functional hydrogel which swells upon some external stimulus^35^.

Here, we demonstrate a new type of barcodes and sensors that integrate all of the key technologies to make cost-effective and mass-produced edible barcodes and sensors. Specifically, silica microspheres are edible and stable, unlike previously used liquid droplets. For the first time, these microspheres are coated with chlorophyll to make them fully edible. CW lasers are used to excite the coated microspheres and observe WGM resonances. Employing multiple monodisperse sizes, which can be mass-produced cheaply, enables the encoding of predefined information and inherent size variation, enabling their use as PUFs. These microspheres can function as free-floating barcodes while simultaneously being a sensor for refractive index. Furthermore, when embedded in edible hydrogel, they can be directly attached to consumable products, remaining insensitive to the environment and stable for extended periods. Finally, embedding it into a functional hydrogel enables its use as a pH sensor.

## Results

### WGM microcavities in the form of chlorophyll-coated silica microspheres

**Fig. 1:**
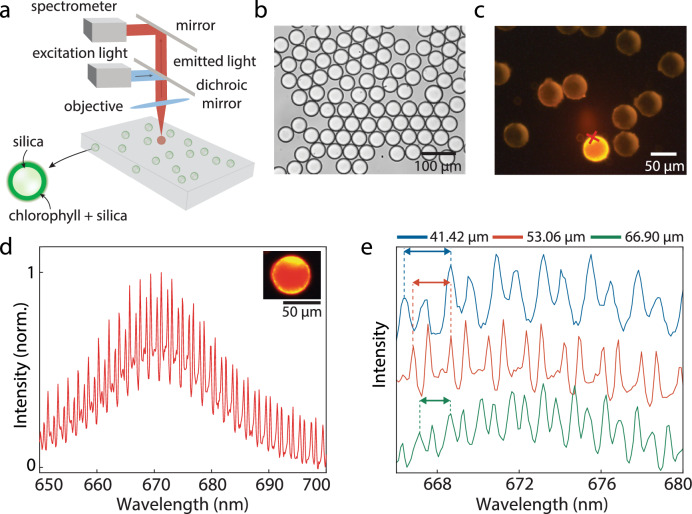
(**a**) Schematics of the experimental setup, which includes chlorophyll-coated silica microspheres and an optical system for the excitation and signal collection. (**b**) Bright-field image of monodisperse silica microspheres with a mean size of 41.78 $\mu \hbox {m}$. (**c**) Chlorophyll-coated silica microspheres with a mean size of 52.10 $\mu \hbox {m}$, one of them is under the excitation of CW laser (highlighted with a red cross). (**d**) Emission spectrum from a single chlorophyll-coated silica microsphere (diameter of 53.06 ± 0.06 $\mu \hbox {m}$) immersed in water. The corresponding microsphere is shown in the inset. (**e**) Comparison of the emission spectra of chlorophyll-coated silica microspheres of three different sizes (41.42 $\mu \hbox {m}$, 53.06 $\mu \hbox {m}$, and 66.90 $\mu \hbox {m}$). The colored arrows indicate the free spectral range for each microsphere, which decreases with increasing microsphere size.

Commercially available monodisperse silica^3^ microspheres with diameters ranging from 40–70 $\mu \hbox {m}$ were used as edible WGM microcavities. The microspheres were coated with a chlorophyll-doped silica layer, followed by a protective silica layer, using the modified Stöber method. The total thickness of the coating ranged from 1−2 $\mu \hbox {m}$. This multilayer coating approach ensured homogeneous incorporation of chlorophyll, minimized leaching, and maintained the surface smoothness of the microspheres. Both chlorophyll extracted from spinach leaves and commercially purchased pure chlorophyll were suitable as gain media, each showing clear WGM resonances. To excite the chlorophyll-coated silica microspheres (Fig. 1a-b), a 450 nm CW laser was used. Under CW laser excitation, the monodisperse chlorophyll-coated silica microsphere emitted light (as shown in Fig. 1c), which was captured and analyzed using a spectrometer. The resulting emission spectrum exhibited a series of distinct peaks corresponding to the WGMs, as shown in Fig. 1d-e. By fitting the emission spectrum using the exact WGM solution, both the cavity diameter and the refractive index contrast between the cavity and its surrounding medium could be determined. Since the refractive index of the silica cavity is known in advance (1.46), we can measure the absolute refractive index of the surrounding medium (see Supplementary material).

We tested three different sizes of chlorophyll-coated silica microspheres, with their emission spectrum presented in Fig. 1e. The measured Q-factors for microspheres with diameters ranging from 40–70 $\mu \hbox {m}$ were between 2,000 and 10,000, while for sizes above 50 $\mu \hbox {m}$ the measured Q-factor was limited by the spectrometer resolution. In our case, the minimum microsphere size was approximately 40 $\mu \hbox {m}$, which was sufficient to support WGM modes. Reducing the size further lowers the Q-factor. However, for microspheres larger than 100 $\mu \hbox {m}$ tend to support high-order WGMs, making spectral analysis more challenging.

To evaluate the photo-degradation behavior of the chlorophyll fluorescent medium under repeated optical readout, measurements were performed using a CW laser with an excitation power of approximately 2 mW focused on a single chlorophyll-coated silica microsphere. As shown in Supplementary Figure 1b, after 2 minutes of continuous laser exposure—corresponding to approximately 250 individual spectral readouts—the peak emission intensity decreased to about 20% of its initial value.

In addition, the long-term stability of the chlorophyll-coated microspheres was evaluated. Chlorophyll-coated silica microspheres prepared approximately 1.5 years earlier were stored in powder form and were re-examined, and were found to still exhibit clear WGM resonances.

### Edible microbarcodes

**Fig. 2:**
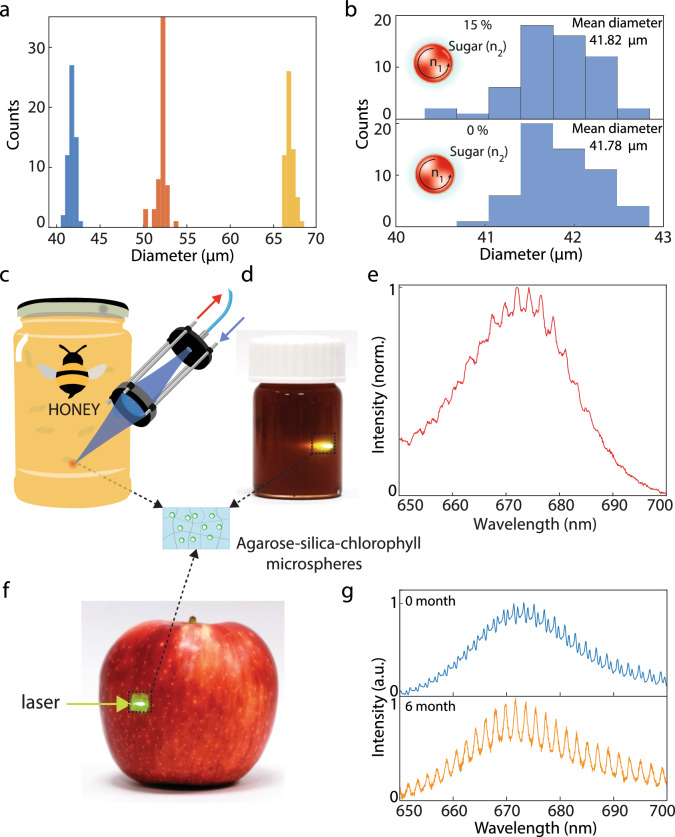
(**a**) Size distribution of chlorophyll-coated silica microspheres from three samples of distinct sizes, obtained by analyzing the emission spectrum of 60 individual microspheres per sample. (**b**) Size distribution of a single sample of chlorophyll-coated silica microspheres dispersed in water at 0 and 15% sugar concentration, showing that the size distribution is independent of sugar concentration. (**c**) illustration of an optical fiber integrated with focusing optics designed to illuminate microspheres and collect their emission in real-time application. (**d**) Agarose matrix containing chlorophyll-coated silica microspheres inside honey bottle, illuminated by a CW laser, and (**e**) emission spectrum collected from agarose matrix. (**f**) Agarose matrix containing chlorophyll-coated silica microspheres attached on an apple surface, illuminated by a green CW laser. (**g**) Emission spectra of chlorophyll-coated silica microspheres inside the agarose matrix at 0 and 6 months, under the excitation of CW laser, note that the agarose film was fully dried before the six-month measurement.

To explore the potential of chlorophyll-coated silica microspheres as barcodes, emission spectra were collected from approximately 60 chlorophyll-coated silica microspheres from each size, with each illuminated separately using a CW laser. The microsphere sizes were determined from their WGM emission spectra, with mean sizes of 41.78 $\mu \hbox {m}$, 52.10 $\mu \hbox {m}$, and 66.89 $\mu \hbox {m}$. The resulting size distributions for all three sizes (Fig. 2a) show coefficients of variation (CV) of 0.45%,0.58%, and 0.48%, respectively, confirming that all three sizes are monodisperse. In this study, we utilized the size of the microsphere as a unique optical barcode, as each size exhibits a distinct emission spectrum. However, this proof-of-concept demonstration was limited to only three sizes, which were commercially available, thus only enabling the encoding of 3 bits of information. However, this approach can be extended to 30 unique sizes within the range 40–100 $\mu \hbox {m}$ without overlap, thereby increasing the information encoding capacity to 30 bits of information.

Chlorophyll-coated silica microspheres can be used directly as barcodes and sensors without embedding them in a supporting matrix when used inside low-refractive-index fluids, such as water. The microspheres can easily be mixed with water-based products or sprinkled on various solid products. Such bare microcavities are sensitive to the change of the refractive index of the surroundings, which changes the spectrum. However, the size and the external refractive index can be extracted from the spectrum independently, so that even if the refractive index change influences the spectrum, the size measured from the spectra remains the same. To actually test the insensitivity to the changes in the refractive index of the surroundings, the chlorophyll-coated silica microspheres were dispersed in water containing sugar concentrations of 0% and 15%. As shown in Fig. 2b, the size distribution remained the same in both media, indicating that the encoding information is not affected by the variation in surrounding conditions.

We note that size-based encoding using individual microspheres is intended for category-level identification, i.e., determining which predefined size classes are present in a given sample. Since microspheres of very similar or identical sizes can occur within a production batch, a single microsphere’s size alone does not constitute a unique identifier. Similar limitations have been noted for semiconductor quantum dots, where nominally identical structures can exhibit indistinguishable spectral signatures^36^. Unique identification at the single-product level is instead achieved through the PUF approach described in the next section, where the combination of multiple microspheres with slightly different sizes within a patch generates a composite signature that is practically irreproducible.

In a practical perspective, the dispersion of chlorophyll-coated silica microspheres in large-volume liquids does not require scanning the entire volume for barcode readout. Owing to their higher density compared to typical food liquids, the microspheres naturally sediment and accumulate near the bottom of the container, allowing barcode readout from a localized region rather than from the full volume. Moreover, the material cost is extremely low: using 5–10 microspheres per barcode, the cost is $< \textrm{US}\$0.01$ per barcode (materials only). Although barcode readout in large-volume liquids was not directly performed in this study, our previous work on droplet-based optical microcavities in food jars demonstrated that microcavity-based barcodes in real food environments can be detected using a compact fiber-based optical setup within a few seconds^3^.

However, in high-refractive-index media such as honey and oils, these bare silica spheres do not function properly, due to the insufficient refractive index contrast between the microsphere and the surrounding medium. To address this issue, the chlorophyll-coated silica microspheres were embedded within a low-refractive-index agarose matrix, providing a suitable refractive index environment for microspheres. In a practical implementation, such agarose matrices containing the coated microspheres can be immersed directly into high-refractive-index consumables, such as honey. The barcode readout can be achieved by a compact setup where both excitation and emission can be guided through an optical fiber and focusing optics, as shown in Fig. 2c. As a proof-of-concept demonstration, an agarose thin film, 2 cm $\times$ 2 cm and 0.2 mm thick, embedding chlorophyll-coated silica microspheres was introduced into a bottle of honey, as shown in Fig. 2d. The corresponding emission spectrum from a single microsphere within the film is shown in Fig. 2e. The film remained stable for at least one month.

Additionally, agarose thin film containing chlorophyll-coated silica microspheres was applied directly to the surface of an apple, as shown in Fig. 2f. To evaluate the long-term stability of the barcode within the agarose matrix, we measured the emission spectra at the initial time (0 months) and again after 6 months, as shown in Fig. 2g.

Already within the first day, the agarose matrix containing the microspheres dried completely. Consequently, only single modes were visible after drying (Supplementary Figure 1c). This behavior is attributed to a reduced refractive-index contrast between the dried agarose film and chlorophyll-coated silica microspheres. TM modes are more sensitive to index contrast and have lower Q-factors; therefore, when the refractive index contrast becomes small, TM modes broaden and are no longer clearly visible, whereas TE modes retain sufficiently high Q-factors to remain observable as distinct peaks. Importantly, during the six-month period, the spectrum and, consequently, the diameter of the microsphere remained unchanged, which is critical for barcoding applications.

### Edible physical unclonable functions

Chlorophyll-coated silica microspheres can also be used as PUFs. Any of the microsphere samples (size) shown in Fig. 2a can be used as a PUF. The slight polydispersity within each sample (mean size ± 0.5 $\mu \hbox {m}$) is employed to generate a unique and random barcode. These barcodes are unclonable, because the size of the individual microsphere can be measured with a precision of 40 nm (estimated by repeated measurements of the same microsphere), but the microspheres can only be produced with a precision of 500 nm. This significant mismatch between measurement resolution and the production of microspheres makes exact replication impossible.

**Fig. 3:**
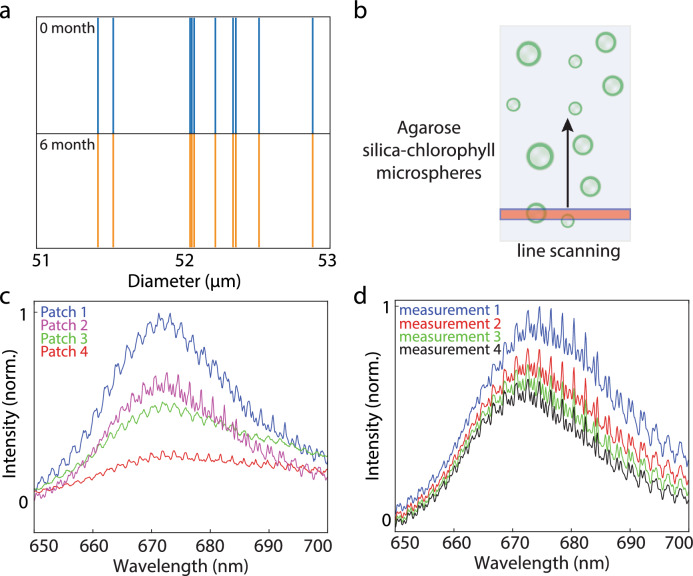
(**a**) Size distribution of chlorophyll-coated silica microspheres within the agarose matrix, measured at 0 and 6 months, by illuminating individual microspheres with a laser. (**b**) Schematics of an agarose patch containing chlorophyll-coated silica microspheres as PUFs, with line scanning used to illuminate the microspheres. (**c**, **d**) Integrated emission spectra from line scans of agarose patches (200 $\mu \hbox {m}$ $\times$ 500 $\mu \hbox {m}$) containing chlorophyll-coated silica microspheres (**c**) Spectra from four different patches, each with 4–5 microspheres, (**d**) Spectra from repeated scans of a single patch with five microspheres, with each spectrum representing one scan.

Here we used a chlorophyll-coated silica microspheres sample with an average size of 52 $\mu \hbox {m}$ as the PUF. Six different agarose patches (samples) were prepared, each containing 5 to 10 chlorophyll-coated silica microspheres. At 0 months, individual microspheres within each sample were manually positioned under the microscope, illuminated using a CW laser, and their emission spectra were recorded to determine their sizes. After six months, the same samples were re-measured using the same procedure. Figure 3a shows the measured microsphere sizes of the one sample at the beginning and after six months; they are identical, demonstrating stability over time. Calculating the sizes of individual beads rather than directly comparing raw spectra allows a clearer assessment of reproducibility. Sizes from additional agarose samples were also determined, and the microsphere size distributions from different samples exhibit clearly distinct patterns, confirming that no two samples produce identical barcodes (Supplementary Figure 2). Each sample is thus characterized by a distinct distribution of microsphere sizes, resulting in stable, unique and non-reproducible PUFs. Using microspheres with a mean diameter of 52 $\mu \hbox {m}$ ± 0.26 $\mu \hbox {m}$ and a measurable size precision of 40 nm, approximately 13 distinct microsphere sizes can be fitted in this size range. The total number of unique PUFs can be estimated as the number of possible combinations $$N_{\textrm{PUFs}} = \left( {\begin{array}{c}n\\ k\end{array}}\right)$$, where $n=13$ is the number of distinguishable microsphere sizes and $k=5$ is the number of microspheres within one patch. This corresponds to approximately $N_{\textrm{PUFs}} =10^{3}$ of unique PUFs. This number can be significantly increased by taking a larger interval of sizes, instead of taking just the relatively monodisperse single sample. If taking the whole usable size range of 40–70 $\mu \hbox {m}$, then the number of measurable unique sizes is 750 and the total number of combinations would be $$N_{\textrm{PUFs}} = \left( {\begin{array}{c}750\\ 5\end{array}}\right) =10^{13}$$.

Although this demonstrated uniqueness, stability, and unclonability fulfill key baseline requirements for PUF behavior, a full quantitative assessment of intrinsic randomness using established statistical metrics, such as the NIST Statistical Test Suite, has not yet been performed. Quantitative studies of chemically produced silica microspheres show stochastic polydispersity and time-evolving size distributions, supporting the inherent randomness exploited here for PUF generation^37,38^. Such an analysis is planned for future work to further substantiate the PUF capability of the microspheres.

To accelerate the scanning process, we employed a line-scanning illumination using a cylindrical lens, which focused the excitation light into a narrow line across the sample (Fig. 3b). The cylindrical lens was used only for illumination, while the emitted light was collected by an imaging spectrometer aligned with the illumination line. This configuration would enable the spectrometer to record spatially resolved spectra along its slit, with one axis corresponding to the spatial position and the other to wavelength. But in our measurements, the recorded spectra were summed along the slit and over time to obtain a single integrated emission spectrum for the whole sample. So the final spectrum is a sum of spectra from all the microcavities in the sample. Such single spectrum contains spectra peaks from multiple beads and is by itself unique and does not require fitting the size of each bead. Although it is possible to acquire a full hyperspectral image by keeping both spatial and spectral information, such an approach requires comparatively more processing time and data handling. Alternatively, our method enables faster measurements while still producing a sufficient number of unique PUFs. Using this method, four samples (agarose patches containing chlorophyll-coated silica microspheres) were scanned. Each scanned patch covered a 2D area of 200 $\mu \hbox {m}$ $\times$ 500 $\mu \hbox {m}$, containing 4–5 microspheres, and the scan was performed at a speed of 0.2 mm $\hbox {s}^{-1}$, the total scan time per sample was 50s. The resulting emission spectra (Fig. 3c) presented the uniqueness of the optical PUFs. Under the same conditions, a single patch was scanned four times at one-minute intervals (Fig. 3d). The emission spectra remained consistent across all scans, indicating good reproducibility. The peak intensity gradually decreased over successive scans due to photobleaching of chlorophyll, and a slight wavelength shift was observed, likely resulting from minor changes in the fluorescent material induced by laser illumination. This fast and repeatable readout could greatly enhance the applicability of these edible PUFs for barcoding applications. In the future, the current setup, which relies on stage movement for sample positioning, could be further simplified by replacing the stage motion with an optical scanning (e.g., galvo mirrors).

### Edible sensors

**Fig. 4:**
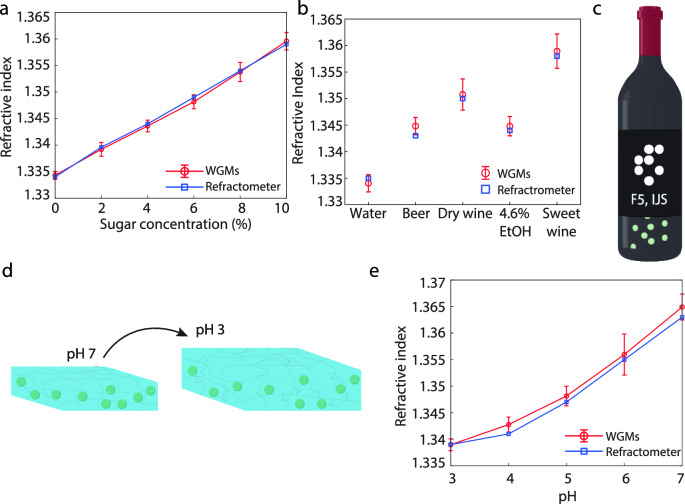
Comparison of refractive index measurements: (**a**) of sugar solutions at different concentrations, and (**b**) across various media using edible microcavities by directly dispersing chlorophyll-coated silica microspheres inside the solutions and measuring with a refractometer. (**c**) Schematic of a wine bottle containing chlorophyll-coated silica microspheres, which can serve as both a barcode and a sensor. (**d**) Schematic of chlorophyll-coated silica microspheres embedded within a chitosan hydrogel matrix at pH 7 and pH 3. As the pH decreases, the chitosan swells progressively, taking up more water and thereby reducing its refractive index. (**e**) Comparison of refractive indices measured using edible microcavities and a refractometer. A chitosan film embedded with chlorophyll-coated silica microspheres was immersed in solutions of known pH, and the refractive indices obtained from both methods showed good agreement.

Next, we employed edible microcavities, specifically their sensitivity to changes in the surrounding medium, to detect crucial factors relevant to food and other consumable products. Chlorophyll-coated silica microspheres were employed as edible optical microcavities to determine the sugar concentration of aqueous solutions by monitoring variations in their refractive index. The microspheres were dispersed in aqueous sugar solutions of varying concentrations. For each concentration, the spectra of five individual microspheres were recorded, and the external refractive index was determined by fitting the emission spectra (Fig. 4a). The resulting refractive index values increased linearly with sugar concentration, resulting in a slope of 0.0031 RIU per % sugar. The average variation among the refractive index values was 0.0013 RIU, with a minimum detectable shift of 0.0007 RIU. Based on slope calibration, these values correspond to sugar concentration uncertainties of 0.42 and 0.23 percentage points, respectively. For validation, the same sugar solutions were measured using a standard refractometer (Fig. 4a). The refractive index values determined from the edible microcavities showed excellent agreement with the refractometer, with an average absolute deviation of 0.001 RIU, which corresponds to only 0.07 percentage points across all concentrations. For reference, commercially available refractometers used in industrial applications, such as measuring sugar in wine, typically with a accuracy of $\pm 0.2$ percentage points. Other substances in food, aside from sugar, could also affect the refractive index. However, in the beverage and other food industries, the refractive index is commonly used to measure sugar concentration. Therefore, our method is compatible with standard measurements in these food industries.

One possible application of our edible sensor is the continuous monitoring of sugar content in sealed beverages, such as sparkling wine or beer, during fermentation. To assess the compatibility of our chlorophyll-coated silica microspheres with the alcoholic environment, the microspheres were directly dispersed in different media, and their refractive indices were measured (Fig. 4b). The edible microcavities showed stable optical emission in these media for over one month. For validation, the refractive indices of the same samples were also measured using an Abbe refractometer, resulting in comparable values (Fig. 4b). For practical implementation, edible microspheres can be embedded within an agarose matrix and attached to the inner surface of the bottle (Fig. 4c) for convenient optical reading or scanning, or left freely suspended within the liquid, allowing them to float throughout the solution. We note that for long-term storage and monitoring applications, such as wine aging processes that can extend over years, further improvement in stability would be required. In such cases, the microspheres could be encapsulated within alternative edible protective matrices, such as food-grade waxes or polymer-based coatings, to avoid coating degradation while preserving sufficient refractive-index contrast to observe WGMs.

Beyond sugar detection, pH monitoring is essential to assess food freshness^39,40^, as changes in pH often indicate spoilage or microbial activity. To address this, we embedded the chlorophyll-coated silica microspheres into a chitosan film. Chitosan is an edible, pH-responsive polymer that swells at lower pH due to increased water uptake, leading to a decrease in its refractive index. To evaluate the sensitivity of chitosan, we immersed a pure chitosan film in solutions of three different pH separately, demonstrating different swelling behavior, as shown in the Supplementary Figure 3b. Subsequently, we tested the chitosan film containing chlorophyll-coated silica microspheres (see Fig. 4d) for pH sensing based on refractive index variations (Fig. 4e). The results reveal a clear decrease in refractive index of chitosan film at lower pH solution, which was validated using refractometer measurements. Our method is capable of estimating the pH of the solution with an error of $\pm 0.3$ pH units, determined from the standard deviation of multiple measurements at each pH. However, swelling of chitosan can be influenced by factors such as ionic strength, multivalent ions, or other chemical components in the surrounding medium. In this study, pH sensing was evaluated in simple buffer solutions, where a reproducible response was observed. For applications in complex food systems, additional calibration or encapsulation strategies may be employed to account for or mitigate the effects of ionic strength and other potential interfering factors. For identifying food spoilage, we are not so much interested in the absolute pH value but in any change within the food. For this reason, the influence of, for example, ionic strength is not so important.

This approach enables real-time pH monitoring of various food products, such as milk and fruit juices. Moreover, these edible sensors offer a non-invasive solution for food safety and quality control and are suitable for integration into packaging. While other packaging sensors often rely on colorimetric dyes to detect parameters such as pH, temperature, or oxygen levels, they are limited by lower sensitivity^41^ and are mostly composed of materials that are not fully edible.

In addition to measuring refractive index changes at equilibrium, monitoring the dynamics of the hydrogel swelling could provide more informative sensing abilities. As the pH-responsive chitosan film absorbs water at lower pH, the refractive index decreases, leading to continuous shifts in the optical resonances of the embedded chlorophyll-coated silica microspheres. The time-resolved tracking of these shifts (Supplementary Figure 3d) shows that the wavelength reaches approximately 80% of its total shift in 10 minutes, and equilibrium is achieved after approximately 20 minutes. This allows direct observation of the swelling kinetics and response times. Previous work has demonstrated similar monitoring of molecular diffusion dynamics in polymer microspheres using optical resonances^42^. Incorporating time-resolved measurements in future experiments could further enhance the functionality and responsiveness of edible microcavity-based sensors.

## Conclusion

In this study, following our previous demonstration of edible microcavity lasers, we explored their operation below the lasing threshold under the excitation of continuous-wave lasers to realize edible optical barcodes and sensors. Importantly, we are now able to produce monodisperse solid microcavities on a large-scale. These edible barcodes and sensors can be applied in direct contact with consumables in various configurations. Their dye stability is significantly enhanced by embedding the microcavities in supporting matrices such as agarose. Moreover, the agarose matrix allows them to be used as barcodes inside high-refractive-index consumables (e.g. honey) and enables direct attachment to solid food surfaces.

As a proof-of-concept, we employed chlorophyll-coated silica microspheres for sugar sensing by directly dispersing them in solution and for pH sensing by embedding them in a pH-sensitive hydrogel. This approach can be further extended to the sensing of other crucial parameters by exploring other naturally available edible sensing materials. Importantly, in all demonstrations the microspheres were excited using CW lasers, providing a simple and low-cost approach, which is suitable for practical use.

In our previous demonstrations, individual microspheres were manually positioned under a microscope, which is impractical. To address this issue, we implemented line-scanning excitation and emission collection, reducing acquisition time and eliminating the need to manually target individual microspheres.

Here, we employed high-resolution spectrometers to demonstrate the proof of concept, as they provide precise control and sensitivity, which were crucial for the initial measurements. However, we acknowledge that these spectrometers are relatively bulky and expensive, potentially limiting practical applications. From a practical perspective, compact, pocket-sized spectrometers with sufficient spectral resolution (0.1–0.2 nm) are commercially available and are capable of resolving the emission peaks of our microspheres. This opens the possibility of developing handheld readers that integrate both a CW laser and a compact spectrometer.

Currently, our work focuses on silica-based edible microcavities, as silica is commercially available at low cost and can be produced as monodisperse microspheres on a large-scale. While silica is approved as a food additive by the FDA and EU, larger silica particles, such as those used in our study, are not specifically covered, and therefore further testing and regulatory approval would be required for direct consumption. Alternatively, these microspheres could be safely incorporated on the inner side of food packaging, where contact with food requires safety but not direct ingestion. Importantly, the methodology developed here is general and can be applied to other edible materials for the creation of microsphere-based barcodes and sensors.

In general, these edible barcodes and sensors are not only limited to food applications but can be extended in the future to pharmaceutical, biomedicine, cosmetics, agriculture, and other high-value consumables.

## Methods

### Materials

Fresh spinach leaves were purchased from a local supermarket for chlorophyll extraction. A 2 g portion of the leaves was washed with hot water, and the veins were carefully removed. The de-veined leaves were placed into a mortar with 1 g of anhydrous sodium sulfate and 4 mL of acetone, and the mixture was ground thoroughly until a smooth paste was formed. The paste was transferred to a 50 mL centrifuge tube. Subsequently, 4 mL of hexane was added, and the tube was gently shaken. An additional 4 mL of distilled water was then added, and the mixture was shaken gently. The mixture was centrifuged until clear phase separation was observed, resulting in a dark green organic layer (top) containing chlorophyll and the bottom aqueous layer consisting of acetone and water. The organic (top) layer was carefully transferred into a new centrifuge tube. To maximize yield, a repeated extraction was performed by adding 2 mL of hexane to the remaining aqueous phase, followed by shaking, centrifugation and collection of top organic layer which was combined with the previously collected organic extract. To remove residual waste, 1 g of anhydrous sodium sulfate was added to the organic phase, stirred, and left to stand for 10 min. The solvent (hexane) was evaporated using a rotary evaporator set to approximately 40 $^{\circ }\hbox {C}$ under reduced pressure. After evaporation, the resulting chlorophyll was dissolved in ethanol for further use.

A stock solution of chlorophyll was prepared by dissolving 1.2 mg of chlorophyll A (USP, reference standard, Rockville USA) in 0.25 mL of ethanol. To this solution, 10 $\mu$L of (3-aminopropyl)triethoxysilane (99%; Merck KGaA, Darmstadt, Germany) and 10 $\mu$L of aqueous nitric acid solution (pH = 1) were added. The mixture was stirred at 600 rpm for 15 minutes in the dark to prevent photodegradation. Separately, 110 mg of silica microspheres were weighed (the same procedure was applied to all three bead sizes obtained from Glantreo and CD Bioparticles) and dispersed in a solvent mixture comprising 6 mL of ethanol, 6 mL of distilled water, and 0.5 mL of aqueous ammonia solution (26 wt.%). Into this dispersion, 125 $\mu$L of the prepared chlorophyll A solution was added, followed immediately by 15 $\mu$L of tetraethoxysilane (TEOS; Merck KGaA, Darmstadt, Germany). The reaction mixture was stirred at 350 rpm in the dark for 6 hours. After the coating process, the chlorophyll-silica-coated microspheres were washed twice with 15 mL of distilled water. The silica-chlorophyll coating process was repeated once more to obtain two chlorophyll-containing silica layers. The multilayer approach was necessary to ensure a sufficiently low concentration of TEOS in each step, thereby minimizing the risk of homogeneous nucleation and the formation of separate silica nanoparticles rather than uniform coating of the silica microspheres. To prevent leaching of chlorophyll, which is incorporated into the silica matrix via electrostatic and hydrophobic interactions rather than covalent bonds, the coated microspheres were further encapsulated with an outer protective silica layer free of chlorophyll. This protective coating was prepared using the same procedure as above, but omitting the chlorophyll solution. Following the final coating step, the chlorophyll-loaded microspheres were washed three times with 15 mL of distilled water.

The silica shell thickness was estimated theoretically based on the amount of TEOS used and experimentally by measuring the microsphere diameters via WGM spectra and optical microscopy. Both approaches yielded consistent thickness of 1–2 $\mu \hbox {m}$, confirming the reliability and uniformity of the coating process.

Agarose film containing chlorophyll-coated silica microspheres was prepared by adding 0.5 g of agarose powder (Fisher Bioreagents, BP160-100) to 50 mL of distilled water, with continuous stirring to prevent clumping. The mixture was autoclaved at 121 $^{\circ }\hbox {C}$ for 20 min. After autoclaving, the solution was allowed to cool approximately 80 $^{\circ }\hbox {C}$ before adding the chlorophyll-coated silica microspheres. The resulting solution was then poured into petri dishes and allowed to solidify at room temperature. The plates were sealed with parafilm to prevent evaporation and stored at 4 $^{\circ }\hbox {C}$. All plates were used within one week of preparation.

Chitosan film containing chlorophyll-coated silica microspheres was prepared by adding 0.2 g of chitosan powder (low-molecular-weight, Sigma Aldrich) to 20 mL of deionized water containing 2 wt% acetic acid. Chitosan was completely dissolved at 80 $^{\circ }\hbox {C}$ in one hour while continuously stirring. The chlorophyll-coated silica microspheres were then introduced into the stirring chitosan solution and dispersed uniformly. The resulting solution was poured into a silicon mold and placed in an oven at 50 $^{\circ }\hbox {C}$ for 12 hours to allow evaporation of water and acetic acid. The dried film was detached from the silicon mold, cut into the required sizes, and washed with ethanol. The final thickness of the chitosan film was in the range of 0.15–0.4 mm.

Refractive-index measurements were performed using an Abbe refractometer (VEB Carl Zeiss) with a precision of ±0.1% Brix. Sugar solutions at different concentrations and chitosan films swollen at different pH values were analyzed. In each measurement, a small amount of sample (solution or swollen film) was placed between the two high-refractive-index prisms of the refractometer. The refractive index was determined by observing the boundary between light and dark fields through the eyepiece, which arises from the refraction of light at the sample–prism interface.

### Optical setup

The samples were observed using an inverted microscope (Nikon Ti2) equipped with a $20\times$/0.45 NA objective. CW lasers at 450 nm and 532 nm were used to excite the microspheres. The laser power on the sample was adjusted between 0.5–4.5 mW using neutral density filters. The emission was collected through an 542 nm long-pass filter. The emitted signal was directed to a high-resolution spectrometer (Andor Shamrock SR-500i, Newton). The spectral acquisition exposure time usually ranged from 0.5–1 s. Bright-field and fluorescence images were recorded using a digital camera (Andor Zyla).

## Supplementary Information

Supplementary Information 1.

## Data Availability

The data that support the findings of this study are available from the corresponding author upon reasonable request.
