# Supplementary material for: Edible optical microcavities for optical barcoding, authentication, and sensing
Source: Sci Rep. 2026 May 7;16:21935. doi: 10.1038/s41598-026-51128-3 (PMC13365461; doi:10.1038/s41598-026-51128-3)
Supplement: Supplementary file 1 — Supplementary Information 1. [file 41598_2026_51128_MOESM1_ESM.pdf]

Supplementary Material
Edible optical microcavities for optical barcoding, authentication, and sensing

Abdur Rehman Anwar, Slavko Kralj, Matjaž Humar

1 Theoretical framework for WGMs

For a spherical microcavity with radius R and effective refractive index n, WGMs arise from the circulation of light in a spherical object due to total internal reflections on the boundary. In a simple geometric approximation, the round-trip optical path length along the cavity circumference is $2\pi nR$. Since WGMs are strongly confined near the cavity boundary, the effective refractive index depends on both the refractive index of the cavity material (internal) and that of the surrounding medium (external). Under this approximation, the resonance condition can then be expressed as:

$$2\pi nR = l\lambda. \tag{1}$$

This approximation provides a quick estimate of the resonance wavelengths, and it is particularly accurate for large polar mode numbers l, where the optical field is tightly confined near the cavity surface. This approximation highlights how WGM resonances depend on the radius of cavity and effective refractive index, but it does not account for polarization, field penetration into the surrounding medium, or the electromagnetic field distribution. To obtain exact resonance wavelengths and field profiles, Maxwell's equations must be solved with appropriate boundary conditions at the cavity interface.

1.1 FSR-based size extraction

In this work, the FSR between two consecutive WGM resonances is used to determine the cavity diameter directly from the experimentally measured spectra. To reduce the experimental uncertainty, the FSR is averaged over multiple neighboring modes of the same polarization,

$$FSR_i = \frac{1}{\lambda_{i-1}} - \frac{1}{\lambda_i}, \quad D \approx \frac{1}{\pi n_{\text{in}} FSR_{\text{avg}}} \tag{2}$$

where D is the cavity diameter, n_{in} is the internal refractive index, take 1.46 for silica, and FSR_{avg} is the average FSR across multiple modes. Here, as a first approximation, we take $n_{\text{eff}} \approx n_{\text{in}}$.

1.2 Exact electromagnetic solution

In spherical geometries, exact solutions to Maxwell's equations yield the eigenmodes of the electromagnetic field under the appropriate boundary conditions at the cavity interface. Each mode is characterized by its polarization (TE or TM) and by the set of three mode numbers. The radial mode number q indicates the number of intensity maxima in the radial direction

inside the cavity. The polar mode number l determines the number of wavelengths fitting along one circulation of the light around the cavity equator. The azimuthal mode number m indicates the inclination of the WGM circulation plane with respect to the reference frame.

For isotropic spherical microcavities, the resonance conditions for TE and TM modes are described by the following characteristic equations:

$$\frac{[n_r x_{nl} j_l(n_r x_{nl})]'}{j_l((n_r x_{nl})} = \frac{[x_{nl} h_l^{(1)}(x_{nl})]'}{h_l^{(1)}(x_{nl})} \tag{3}$$

$$\frac{[n_r x_{nl} j_l(n_r x_{nl})]'}{n_r^{(2)} j_l((n_r x_{nl})} = \frac{[x_{nl} h_l^{(1)}(x_{nl})]'}{h_l^{(1)}(x_{nl})}. \tag{4}$$

Here $n_r = n_{in}/n_{ext}$ is the relative refractive index between the resonator n_{in} and the surrounding medium n_{ext}. The x_{nl} is the size parameter, expressed as $x_{nl} = n_{ext} \cdot \omega_{nl} \cdot R/c$, where ω_{nl} is the angular eigenfrequency, R is the radius of the resonator and c is the speed of light. The functions j_l and h_l denote the spherical Bessel and Hankel functions, respectively, and the primes indicate differentiation with respect to their arguments. Numerical solution of these characteristic equations gives the exact resonance frequencies and electromagnetic field distributions for both TE and TM modes. For each polar mode number (l), multiple solutions exit, corresponding to different radial mode numbers (q). Each is associated with a complex eigenfrequency (ω_{nl}), reflecting the fact that resonant modes continuously dissipate their energy through radiation into the surrounding medium.

1.3 Asymptotic approximation and mode identification

Although exact electromagnetic solutions are accurate, they are computationally intensive. For high-Q microcavities with large polar mode numbers (typically on the order of 100) and low radial mode numbers, the optical field is strongly localized near the cavity boundary ($r = R$). In this regime, asymptotic approximations offer an efficient and accurate alternative by using analytic expansions of the Bessel functions. The resonant frequencies can then be expressed as an asymptotic series in powers of $(l/2)^{-1/3}$ [1,2].

$$n_{in} kR = l - \alpha_q \left(\frac{l}{2}\right)^{1/3} - \frac{\chi n_r}{\sqrt{n_r^2 - 1}} + \frac{3\alpha_q^2}{20}\left(\frac{l}{2}\right)^{-1/3} - \frac{\alpha_q n_r \chi (2\chi^2 - 3n_r^2)}{6(n_r^2 - 1)^{3/2}}\left(\frac{l}{2}\right)^{-2/3} + \mathcal{O}(l^{-1}) \tag{5}$$

Here, R is the cavity radius, k is the wavenumber, and χ depends on polarization: $\chi = 1$ for TE modes and $\chi = 1/n_r^2$ for TM modes. The polar mode number satisfies $l \gg 1$, and $q = 1, 2, 3, \ldots$ is the radial mode number; the approximation becomes less accurate for higher radial modes ($q > 1$). The parameter α_q denotes the q-th zero of the Airy function corresponding to the radial mode.

In this work, the asymptotic approximation is first used to obtain an initial estimate of the external refractive index (n_{ext}) by comparing calculated TE and TM mode positions with experimentally observed resonances. This polarization-dependent formalism provides the basis for assigning TE and TM mode numbers in the measured spectra (Supplementary Figure 1a). Once an optimal value of the external refractive index is identified, the resonance wavelengths are refined by numerically solving the characteristic equations (Eqs. (3) and (4)), which provides higher accuracy than the asymptotic model (Eq. (5)) alone. By combining this fitted refractive index with the cavity diameter extracted from the experimental FSR, the method enables simultaneous determination of both the cavity size and the refractive index of the surrounding medium. Notably, repeated fitting of multiple spectra from the same microsphere yields a mean diameter of 52.01 µm with a standard deviation of ~40 nm, while the corresponding WGM peak positions vary by only 0.05-0.10 nm.

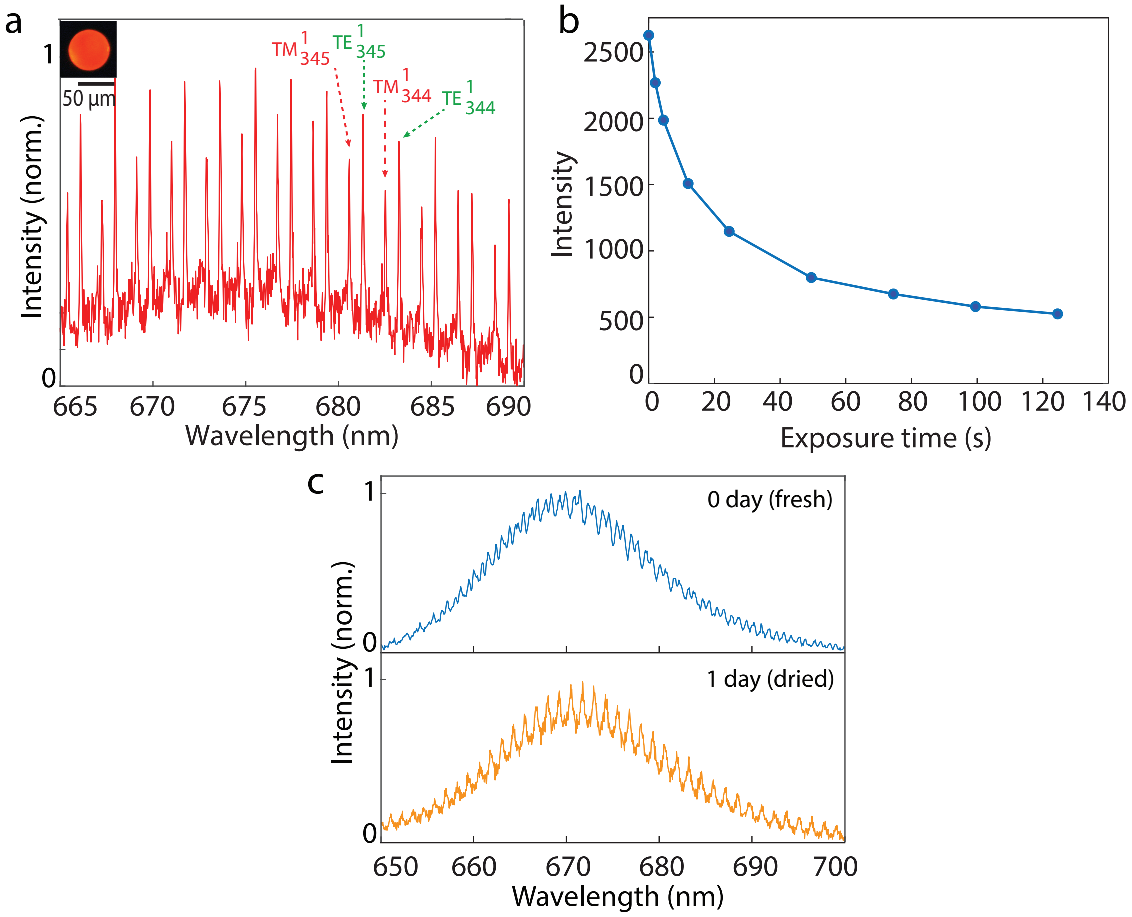

Supplementary Figure S1: *a) Emission spectrum of a single chlorophyll-coated silica microsphere under the excitation by a blue CW laser. b) Peak emission intensity of a chlorophyll-coated silica microsphere with respect to laser exposure time, showing progressive photodegradation during repeated optical readout. c) Emission spectra of chlorophyll-coated silica microspheres inside the agarose matrix at 0 and 1 day, under the excitation of CW laser.*

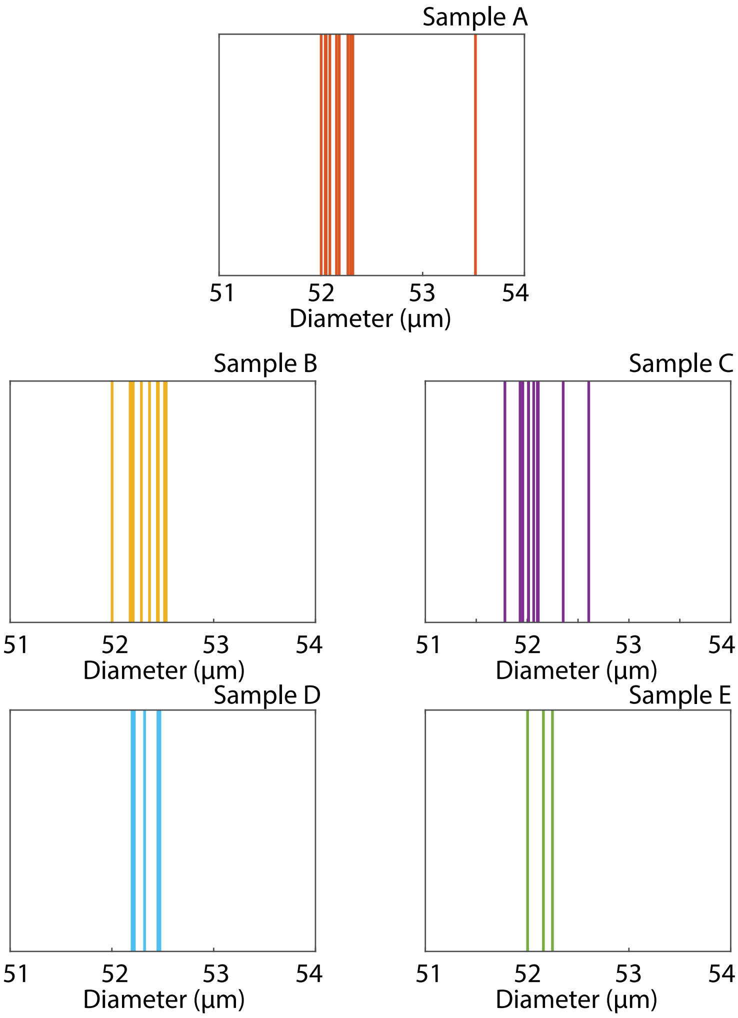

Supplementary Figure S2: *Size distribution of chlorophyll-coated silica microspheres within the agarose matrix at 0 months. Measurements were performed on five different samples by illuminating individual microspheres with a CW laser.*

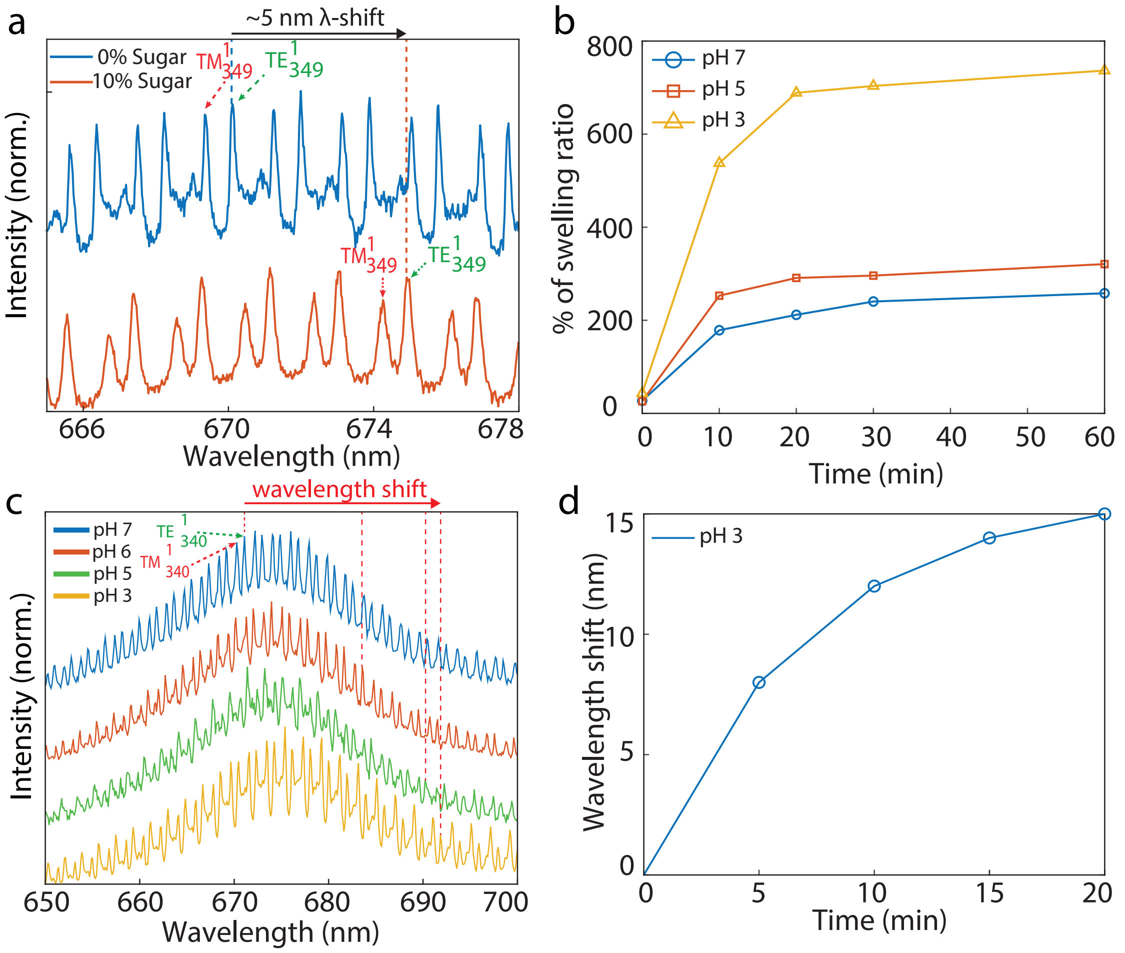

Supplementary Figure S3: *a) Emission spectra of chlorophyll-coated silica microspheres dispersed in two different sugar solution concentrations; the wavelength shifts were used to determine an optical sensitivity of* 208 nm/RIU*. b) Chitosan film immersed in various pH solutions, with its weight measured at different time intervals, and the swelling behavior was significantly pH-dependent. c) Emission spectra of chlorophyll-coated silica microspheres embedded within a chitosan film were measured while immersed in solutions of different pH. An average pH sensitivity of approximately* 5.7 nm/pH *was observed over the pH range from 7 to 3. Notably, the highest sensitivity occurs between pH 7 and pH 6, reaching approximately* 13.7 nm/pH*. As the pH decreases further below pH 5 , chitosan swelling approaches saturation, resulting in a reduced sensitivity. d) Continuous monitoring of the emission wavelength shift of a chlorophyll-coated silica microsphere embedded in chitosan, showing the dynamic response from equilibrium to pH 3.*

References

[1] CC Lam, Peter T Leung, and Kenneth Young. Explicit asymptotic formulas for the positions, widths, and strengths of resonances in mie scattering. *Journal of the Optical Society of America B*, 9(9):1585–1592, 1992.

[2] S Schiller. Asymptotic expansion of morphological resonance frequencies in mie scattering. *Applied optics*, 32(12):2181–2185, 1993.
